# Correction: Gastrointestinal tract disorders classification using ensemble of InceptionNet and proposed GITNet based deep feature with ant colony optimization

**DOI:** 10.1371/journal.pone.0297737

**Published:** 2024-01-22

**Authors:** Muhammad Ramzan, Mudassar Raza, Muhammad Irfan Sharif, Faisal Azam, Jungeun Kim, Seifedine Kadry

There are errors in the Funding section. The correct Funding statement is: This research was partly supported by the Technology Development Program of MSS (No. S3033853) and by the research grant of the Kongju National University in 2023.
